# Train the trainer course for general practice trainers in ambulatory care: the Berlin model

**DOI:** 10.3205/zma001321

**Published:** 2020-04-15

**Authors:** Ulrike Sonntag, Antje Koch, Gudrun Bayer, Christoph Heintze, Susanne Döpfmer

**Affiliations:** 1 Charité – Universitätsmedizin Berlin, Institut für Allgemeinmedizin, Kompetenzzentrum Weiterbildung Berlin, Berlin, Germany; 2 Ärztekammer Berlin, Berlin, Germany

**Keywords:** train the trainer, postgraduate medical specialty training, didactic, competence, training concept

## Abstract

**Objective: **Demands for a stronger competence orientation of specialty postgraduate medical training require the expansion of the didactic qualifications of those responsible for postgraduate medical training. In the context of the foundation of the Berlin competence center for postgraduate general practice training, a train the trainer basic seminar was designed together with the Berlin chamber of physicians. The seminar aims to convey formal-legal aspects in close connection with the development of didactic competences of the general practice trainers. This article presents the didactic concept, focal points and the schedule of the one-and-a-half-day seminar to be able to adapt it to one's own context.

**Methodology: **After the seminars, participants filled out an evaluation form. The questionnaire included the subjective experiences of increased competence, the relevance of the contents, and the satisfaction with the structure and methods of the seminar. The data were analyzed descriptively.

**Results: **Since June 2018, 46 general practice trainers have participated in one of three train the trainer seminars. 97.6% of the participants were very satisfied or satisfied with the overall seminar and felt that the timeframe was right, 92.7% would recommend the seminar to colleagues. 68.3% fully agreed that by attending the seminar they were able to improve their didactic skills, 90% were confident that they could integrate what they had learned into their work as general practice trainers. 85.4% stated that they had reflected on their role as trainers. In particular, the atmosphere, the high degree of interactivity and the protected framework for collegial exchange were positively emphasized.

**Conclusion:** Together with the Berlin chamber of physicians, we succeeded in designing a train the trainer seminar which, on the one hand, met the needs of the general practice trainers for clarification of formal-legal questions of their further training activities and, on the other hand, allowed the further development of didactic skills. At the moment, a modular program is being planned in order to give general practice trainers the opportunity to expand their didactic competence and regularly exchange ideas with colleagues.

## Zusammenfassung

**Zielsetzung: **Forderungen nach einer stärkeren Kompetenzorientierung der Facharzt-Weiterbildung erfordern den Ausbau didaktischer Qualifikationen der Weiterbildungsbefugten. Im Rahmen der Gründung des Berliner Kompetenzzentrums Weiterbildung wurde gemeinsam mit der Landes-Ärztekammer ein Train the Trainer-Basis-Seminar für Weiterbildungsbefugte in der ambulanten Versorgung konzipiert. Ziel des Seminars ist die Vermittlung formal-rechtlicher Aspekte in enger Verzahnung mit dem Ausbau didaktischer Kompetenzen der Befugten. Das didaktische Konzept, Schwerpunkte und der Zeitplan des anderthalbtägigen Seminars werden dargestellt, um es für den eigenen Kontext adaptieren zu können.

**Methodik: **Im Anschluss an die Seminare füllten die Teilnehmenden einen Evaluationsbogen aus. Erfragt wurde der subjektiv erlebte Kompetenzzuwachs, die Relevanz der Inhalte und die Zufriedenheit mit der Struktur und den Methoden der Veranstaltung. Die Angaben wurden deskriptiv ausgewertet.

**Ergebnisse:** Seit Juni 2018 haben 46 Befugte an einem von drei Train the Trainer-Seminaren teilgenommen. 97,6% der Teilnehmenden waren mit der Gesamtveranstaltung sehr zufrieden bzw. zufrieden und empfanden den zeitlichen Rahmen als genau richtig, 92,7% würden die Veranstaltung Kolleg*innen weiterempfehlen. 68,3% stimmten voll zu, dass sie durch den Besuch des Seminars ihre didaktischen Fertigkeiten ausbauen konnten, 90% waren zuversichtlich, dass sie Erlerntes in ihre Tätigkeit als Befugte integrieren können. 85,4% gaben an, ihre Rolle als Weiterbildungsbefugte reflektiert zu haben. Insbesondere die Atmosphäre, der hohe Grad an Interaktivität sowie der geschützte Rahmen zum kollegialen Austausch wurden positiv hervorgehoben.

**Schlussfolgerung: **Es ist gelungen, gemeinsam mit der Landes-Ärztekammer ein Train the Trainer-Seminar zu konzipieren, welches einerseits den Bedürfnissen der Befugten nach Klärung formal-rechtlicher Fragen ihrer Weiterbildungstätigkeit gerecht wurde als auch den Ausbau didaktischer Fertigkeiten realisieren ließ. Momentan wird ein modular strukturiertes Aufbau-Programm geplant, um Befugten in regelmäßigen Abständen die Gelegenheit zum didaktischen Kompetenzausbau und kollegialen Austausch zu geben.

## Introduction

Based on the Care Improvement Law (Section 75a SGB V), competence centers (KW) for postgraduate general practice training have been established nationwide since 2017 to support the further training of general practitioners (GP) [https://www.bundesgesundheitsministerium.de/service/begriffe-von-a-z/g/gkv-versorgungsstaerkungsgesetz.html]. The connection of most of the centers to university departments of general practice offers the opportunity to link student training and specialty medical training. In addition to seminar and mentoring programs for physicians in postgraduate training (ÄiW), KW also offer train the trainer (TtT) seminars for specialists in ambulatory care. At the Berlin KW (located at the Institute of General Practice, Charité – Universitätsmedizin Berlin) general practice trainers, who ensure further clinical training in outpatient care, receive didactic and methodological training. Legal, formal and organizational questions concerning the role of trainers are also addressed. To convey all these aspects, close coordination of the conception and implementation of the seminars with the respective chamber of physicians (Berlin Chamber of Physicians – LÄK) is advisable. Both KW and LÄK design and carry out the Berlin TtT seminar. The central feature of the concept is the close interlinking of formal and didactic aspects.

Up to now, only formal criteria are required to obtain an authorization for postgraduate medical training of future GPs [1], [https://www.aerztekammer-berlin.de/10arzt/15_Weiterbildung/16_Fragen_Antworten/17_02Weiterbildungsbefugnis/index.html]. In contrast to common practice in other European countries [2], no didactic knowledge is required. The TtT seminars presented here aimed to impart knowledge and skills relevant to trainers.

## Project description: the Berlin model – train the trainer

Short workshops for didactic qualification are resource-saving. Personnel and planning costs are low, and the participants save time and money [3]. Nevertheless, the benefits of workshops are controversial, because they can hardly have a long-term effect as a single event [4]. So far, there are only a few comprehensible descriptions of successfully evaluated training models, as highlighted by Bylund et al. [5]. Our study helps to close this gap and aims to describe a TtT seminar for outpatient GP trainers. 

In 2018, KW Berlin designed a TtT seminar for general practice trainers. It was based on the recommendations of the TtT working group of the section for postgraduate medical training of the German College of General Practitioners and Family Physicians – DEGAM [6], the experiences of the KW Baden-Württemberg [1], [7] and many years of our own experiences teaching physicians for student training.

The close cooperation with the LÄK was central to the development of the content and didactic concepts, to interlink didactic and formal-legal questions concerning postgraduate medical training appropriately. In addition to teaching the regulations of the LÄK (for example, granting a training license, the postgraduate training curriculum, further training discussions, checking the achieved and not achieved training objectives, the preparation of certificates and filling out the logbooks as specifications of the further training regulations), the seminar focuses on the concrete implementation of these specifications in practice. Additionally, the Association of Statutory Health Insurance Physicians (Kassenärztliche Vereinigung Berlin – KV) was involved in the conception of the seminars. Cases were developed in advance and commented on by the state KV. The cases concerning relevant legal requirements in the context of specialty training, such as interruption of training, illness of the trainers or leave of absence for further training, were initially dealt with in small groups in the seminar and then discussed in plenary sessions. 

ÄiW were also involved in the planning and implementation of the seminars. The regional group Weiterbildung Allgemeinmedizin Berlin (WABE), an offshoot of Junge Allgemeinmedizin Deutschland (JADE), interviewed Berlin ÄiW regarding wishes, suggestions and criticism of the current situation of their training. Core statements, for example, that ÄiW would like to see a structured initial training by GP trainers and regular feedback meetings, were presented and discussed at the seminar.

In contrast to similar seminars offered at other locations, the basic seminar described here is aimed at all licensed trainers for outpatient care. Therefore, it is not only suitable for general practitioners, but also for orthopedic surgeons in ambulatory care and other outpatient specialists who train future general practitioners. 

### Schedule and didactic concept

The basic seminars consisted of 15 teaching units of 45 minutes, spread over two consecutive days. Following the needs of the target group, a Friday afternoon and the following Saturday proved to be convenient to allow for consultation hours and subsequent seminar attendance. 

The Berlin TtT seminar followed the chronological order of the tasks arising before, during and after every postgraduate training section: from the conception and implementation of a curriculum adapted to needs of the practice, through recruitment, hiring and contract design, familiarization and supervision of ÄiW as well as conflict and feedback discussions, to tasks within the framework of the documentation of postgraduate specialty training. These topics served as a common thread in the seminar and were the basis for the agenda.

Before the seminar, participants were asked to reflect on their role and in particular on the strengths and weaknesses of their post-graduate training activities in an online survey. For this purpose, the CanMeds questionnaire of KW Baden-Württemberg was used [7]. This preliminary survey was used to prepare the participants for the topic and to tailor the contents of the seminar to the needs of the participants. According to theories of adult education, a decisive factor for learning success is the connection to experience and previous knowledge of the participants [8]. The results of the preliminary survey were consulted at various points during the seminar. After the seminar, the participants received detailed photo documentation, a folder with all important information, forms and checklists for postgraduate training, and further support in the implementation of their self-defined goals. 

The seminars were characterized by a high degree of participant activity. The contents were jointly developed and, in some cases, directly tested. Didactically, we worked primarily with simulations, case discussions and small group methods that encouraged reflection and exchange. Following the recommendations of the DEGAM TtT checklist [6], an integral part of the seminars was the exchange at peer level between GP trainers who worked together in varying small group configurations. A lecturer from KW and a representative of LÄK was involved throughout the seminar, and two other lecturers from KW were present on the Saturday morning to moderate small group sessions. A detailed description of the seminar procedure, the methodological implementation and the combination of didactic and formal-legal contents can be found in attachment 1 .

#### Focus of the seminar 

One focus of the seminar was the design of curricula for postgraduate medical training based on the individual conditions of the respective practice. The LÄK in Berlin demands the submission of a postgraduate training curriculum within the scope of the authorization, for which minimum requirements are specified. The practical design of curricula varies greatly. According to the experience of the LÄK, detailed plans are submitted in part, but also rather superficial curricula, which correspond to the formal criteria but hardly contribute to the structuring of the postgraduate training section. Patterson et al. [9] recommend designing competence-based curricula that prepare for work in outpatient care. The structuring of postgraduate specialty training based on catalogs has often been criticized [10]. A competence-based curriculum for postgraduate specialty training in general practice exists for German-speaking countries [11]. This was developed by the participants themselves in the seminar, and was based on the CanMeds model [12]. Based on a case vignette, the participants generated ideas for structuring the postgraduate specialty training and developed a schedule for a 12-month outpatient training period. 

In addition to other TtT seminars, we focused on the presentation of the entrusted professional activities (EPA) concept, which is internationally recognized and supports the structuring of postgraduate specialty training [13], [14], [15], [16]. In the seminar, participants exchanged their views on questions concerning the supervision of the ÄiW and the increasing degree of independence of the ÄiW which is aimed for in a postgraduate training section. Ideas were presented on how the degree of independence can be measured and what possibilities exist for making so-called "entrustment decisions" [17], [18]. 

Another focus of the seminar was the practical training of communication in different situations with ÄiW. For this purpose, GP trainers and ÄiW created specific cases for simulated training and actors were trained. A challenge in the conception of the cases was the identification of general communicative competencies for the work of GP trainers, that can be transferred to other situations. In the seminar, the participants experienced three different conversational situations, which they tested in role-plays in small groups and evaluated afterward. Each case lasted approximately 60 minutes, and in addition to the actual simulation, the focus was on detailed and structured evaluation using observation tasks [19].

##### The cases in detail:

One simulated conversation was a feedback talk with an ÄiW, in which the challenge was to give positive feedback regarding the general performance, but also report that she/he was very insecure and asked too many questions. Huenges et al. report that subjectively experienced insecurity in postgraduate specialty training, especially during the transition from inpatient to outpatient postgraduate training, is a major challenge [20]. This simulation was prepared together with the participants by collecting content and structuring aids for these simulated conversations. 

In a second simulated conversation, the GP trainer dealt with a request from an ÄiW for short-term leave of absence for another further training lasting several days. Here, the focus was on the much-cited generation conflict and the discussion of Generation Y. Much room was given to the subsequent discussion about the heterogeneous handling of the demands of the ÄiW. 

The third simulation required giving feedback to an ÄiW after an unsuccessful patient interaction. KW Baden-Württemberg kindly provided us with this case. 

## Methodology

Directly after the seminars, the participants filled out an evaluation form with 23 items with a five-point-scale (1=fully agree – 5=disagree, 3 items) and a three-point-scale answer format (1=agree – 3=disagree, 20 items). Sociodemographic data and free-text information regarding the evaluation of the seminar and further training needs were also recorded. The evaluation was intended to ascertain whether the participants had, in their estimation, achieved the learning objectives and intended skills, whether they found the content relevant, and whether they were generally satisfied with the structure and methods of the course. The data were evaluated descriptively using SPSS 24.0. 

## Results

Since June 2018, 46 GP trainers have participated in one of three TtT seminars. Evaluation forms are available for 41 participants. On average, the participants (55% male, 45% female) were 54 years old at the time of the seminar (SD=7.64). The GP trainers completed their speciality examination between 7-40 years ago (median 18 years). Participants had trained ÄiW for a median of seven years (range 0-27 years). 87.2% of the participants had their own practice, 10.2% were employed. 78.9% worked together with other teaching staff in a joint practice or professional association. The participants stated that they worked on average 42.79 hours per week (SD=12.48). In the last five years, the participants trained a median of four ÄiW (range 0-15). 

Motivations to participate in the seminar were: the desire to learn new things; to be better able to fulfill their role as GP trainers; to be able to clarify concrete questions about dealing with the ÄiW; to explore legal and organizational aspects of further training. 

97.6% of the participants were very satisfied or satisfied with the overall event and felt that the time frame was just right, 92.7% would recommend the event to colleagues. 84.6% wished for further TtT seminars on didactic topics, legal issues and communication challenges as trainers. A combination of didactic training with general medical topics was suggested. 68.3% fully agreed that by attending the seminar they could improve their didactic skills, 29.3% partially agreed. 85,4% of the participants stated that they had reflected on their role as GP trainers, the remaining 14.6% partially agreed. 90% of the participants were confident after the seminar that they would be able to integrate what they had learned into their work as trainers. Further evaluation results are shown in table 1 (Tab. 1). 

In free-text comments, the atmosphere, the high degree of interactivity and activity in the seminar and the protected framework for collegial exchange were particularly emphasized. Some participants stated that they had critically questioned and partly redefined their role as trainers through the seminar. 

## Discussion

The special features of the Berlin TtT (the agenda along the chronological sequence of trainers’ activities, the intensive communication training in small groups, the inclusion of the perspective of the ÄiW, the consistent participative conception and implementation together with the Berlin chamber of physicians as well as the integration of the EPA concept into the qualification) were very well received. The space for participation and exchange among the participants was particularly beneficial. 

The evaluation focused on the self-assessed increase in competence and satisfaction with the seminar. This covers the two lower evaluation levels of the four-level model of evaluation according to Kirkpatrick [21]. Concepts for checking the sustainable effectiveness and the actual increase in competence must be developed to be able to prove the benefit of such interventions with objective criteria. 90% of participants assumed that they would be able to put what they learned into practice. This results speak not only for the quality of the seminar but also the high motivation and self-efficacy expectations of the participants. According to Bandura [22], these are essential factors for the successful implementation of what has been learned. 

Steinert et al. [23] identified success factors of didactic qualifications, which were taken up in the conception of the Berlin TtT seminar: the participants tried out new things in the protected seminar framework and planned the transfer into everyday life, various methods promoted the activation of the participants and stimulated the exchange of ideas among colleagues. Especially in the context of simulating various occasions for talks with ÄiW, an intensive and sometimes controversial exchange among the GP trainers took place, which was perceived as very profitable by the participants. The selected simulated cases were very well suited to stimulate reflection and critical examination of the trainers’ role. The relatively large amount of time and structured framework for the simulations allowed for a more in-depth discussion and the identification of a wide range of options for action. 

The positive feedback from the participants and the many formal, organizational and legal questions concerning postgraduate specialty training show that the joint implementation of the seminars with the LÄK is very profitable. The inclusion of the Association of Statutory Health Insurance Physicians, as already practiced in other federal states, can also bring added value for the participants. 

Many of the participants were teaching doctors from the Institute of General Practice and had already completed didactic training in this role. Only 68.3% of the participants fully agreed that the seminar enhanced their didactic skills. One explanation could be that most of our participants were didactically pre-qualified. On the other hand, due to the close interlocking of didactic and formal topics, it is not always clear to the participants which elements served the didactic qualification. Perhaps the participants tended to underestimate the actual increase in competence at the end of the seminar. Follow-up surveys are in preparation and may clarify these questions.

With our seminar, we first reached the already committed GP trainers who were interested in the quality of postgraduate specialty training. As an incentive for participation, we offer to mention the participants on the KW homepage. Given the lack of young doctors and the increasing difficulty in recruiting ÄiW, this may motivate more to participate in future. We also plan to document seminar participation in the LÄK's list of GP trainers. Van Dongen et al. [24] showed that the publication of participation leads to significantly more physicians participating in a voluntary qualification. Whether this intervention is effective for participation in TtT seminars cannot be said with certainty now. In England, Denmark and the Netherlands, GP trainers must participate in considerably longer TtT seminars, further quality assurance measures and advanced training courses in order to obtain or retain a license [2]. It should be discussed whether it ought to be obligatory for all GP trainers to participate in TtT seminars to strengthen the quality of GP training.

Outpatient GP trainers have few opportunities for structured exchange and emphasize this aspect very positively in the evaluation of the seminar. It is important to develop structures in which they can meet regularly to discuss their role as GP trainers.

When designing TtT seminars it is important to take the perspective of the ÄiW into account. Our approach of asking the ÄiW in advance what they wanted from their trainers was pragmatic and resource-saving, which lead to the seminar addressing topics relevant to young primary care physicians. From time to time, ÄiW were also present at the seminar and could bring in their perspective. It is planned to further promote the exchange between trainers and trainees and to invite ÄiW to the seminars for a certain time. In Denmark, the structured exchange with ÄiW is an integral part of the TtT seminars [2]. However, most of the seminar time should be held without ÄiW to ensure open and protected exchange among the trainers. 

Doctors' time resources are limited, so it is important to make effective use of their time in the seminar and to provide the trainers with practical tools that they can apply directly. In the future, blended learning approaches could be used more often to make optimal use of available time. Our experience with an online preliminary survey of the participants was very positive, with 90% of the participants taking the time to reflect on their role as trainers. This saves time in the seminar, which is then available for practical testing of methods, reflection and discussion with others.

## Conclusion

Together with the LÄK, a TtT seminar was successfully designed which, on the one hand, met the needs of the GP trainers for clarification of formal and legal questions and, on the other hand, allowed the development of didactic skills. The didactic concept is also transferable to other target groups: the LÄK, for example, has implemented it and offers TtT seminars for trainers in inpatient settings. 

Topics for advanced TtT seminars have been identified and will be offered for the first time next year. A modular advanced program is planned. Graduates of the basic module can develop their didactic skills in various topics, and the exchange of ideas on current issues with colleagues should also be a feature of this program. The advanced training courses are planned as afternoon events with the possibility of a joint dinner afterward. Planned topics of the advanced modules are peer counseling on difficult situations as GP trainers, a feedback refresher, the development of EPAs for outpatient training, teamwork, and conducting job interviews.

In the future, it will be necessary to develop criteria that prove the sustainable effectiveness of the program. A follow-up survey of the participants is in preparation and may provide valuable information for the further development of the program. 

The support of the postgraduate specialty training of future GPs within Care Improvement Law and the associated promotion of the KW can serve as an example for other specialty training programs. General practice plays a pioneering role in this important and necessary development. It provides concepts for the structuring and interlinking of undergraduate and postgraduate education, didactic qualification of the trainers, as well as supporting the ÄiW with a seminar and mentoring program. 

## Acknowledgements

We would like to thank KW Baden-Württemberg for the support and the collegial exchange of ideas in the conception of the Berlin train the trainer seminar. 

## Competing interests

The authors declare that they have no competing interests. 

## Supplementary Material

Content and time schedule of the train the trainer seminar, division of topics between KW and LÄK

## Figures and Tables

**Table 1:**
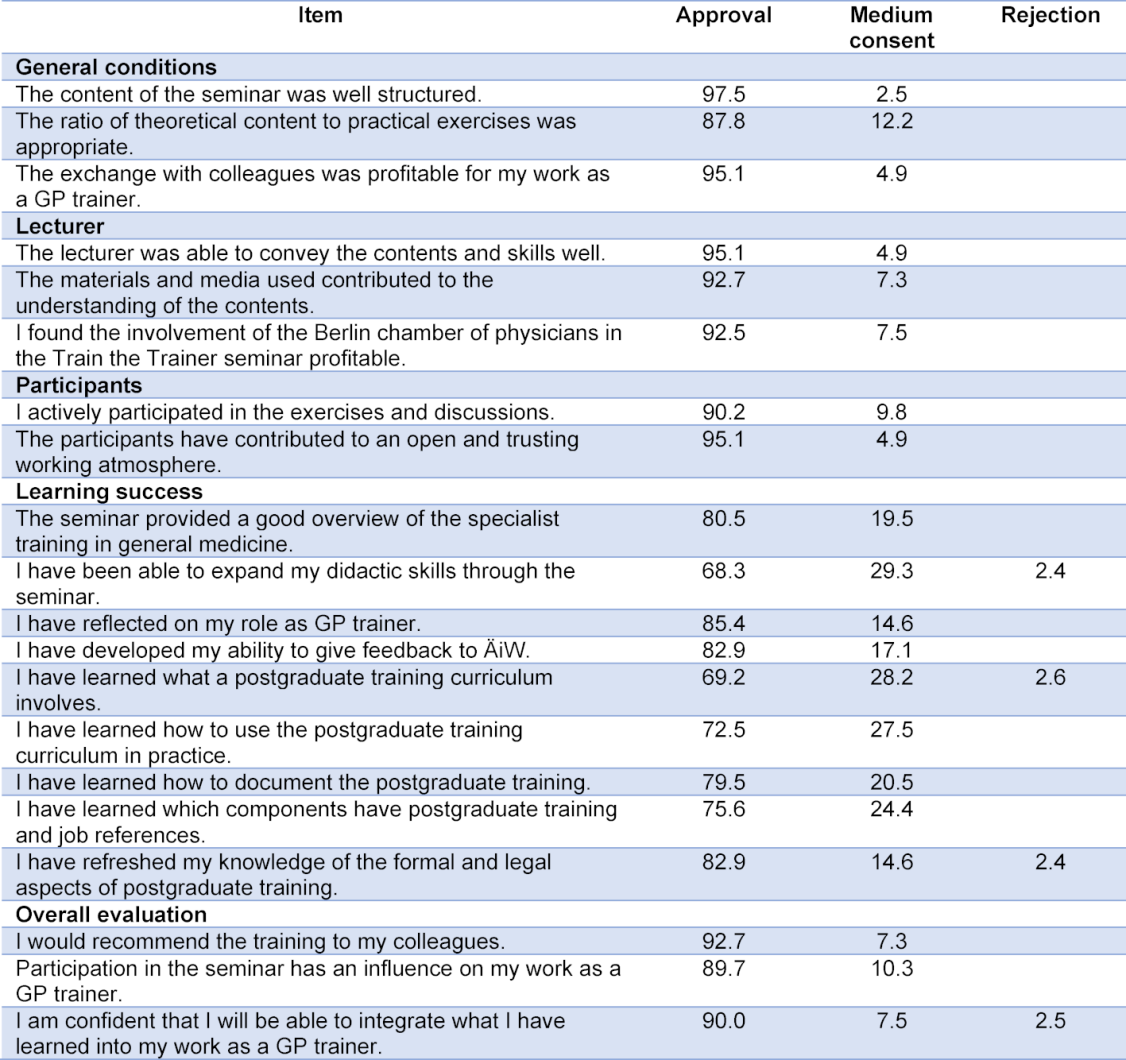
Evaluation of train the trainer seminars, n=41, information in percent
