# Supplementary material for: Train the trainer course for general practice trainers in ambulatory care: the Berlin model
Source: GMS J Med Educ. 2020 Apr 15;37(3):Doc28. doi: 10.3205/zma001321 (PMC7291383; doi:10.3205/zma001321)
Supplement: Content and time schedule of the train the trainer seminar, division of topics between KW and LÄK [file JME-37-28-s-001.pdf]

Attachment 1: Content and time schedule of the train the trainer seminar, division of topics between KW and LÄK

Content	Methods	Time in min	Actors
**Before the seminar as online task: reflection on one's own role as a GP trainer, analysis of strengths and weaknesses, expectations**			
**Friday afternoon 15-19**			
Welcome by lecturers from KW, director of the Institute of General Practice and LÄK and presentation of KW and its fields of activity		15	KW and LÄK
Get to know	Setting up	15	KW
Recourse to online task	Presentation of results and discussion	15	KW
Present agenda, present materials	Common thread: the path of the ÄiW through the postgraduate specialty training	5	KW
Why are we offering TtT seminars?	Lecture and discussion, figures on the lack of GPs, specialty qualifications etc.	15	LÄK
What constitutes good specialty training?	Activation of the participants through brainstorming, 3 corners in the room: the ideal ÄiW; the ideal trainer; the ideal place for specialty training	15	KW
What does specialty training mean?	Continuing education regulations, logbook, obligations of trainers, criteria for licensing, specifications for the specialty training curriculum	10	LÄK
**Break (15 min)**			
**Common thread: ÄiW is not yet in the practice - what tasks do licensed trainers have in advance?**			
**Curriculum of postgraduate specialty training**	a) Think-pair-share: what can you teach an ÄiW?<br>b) Recourse to preliminary questioning<br>c) Presentation of the CanMeds-roles by lecturers<br>d) Classification of the collected aspects in CanMeds by participants<br>e) Development of a timeline for a 12-month training period in small groups and presentation in plenary<br>f) Introducing EPA's concept by means of examples - exchange on delegation of responsibility and autonomy in small groups/in plenary<br>g) What is already available: e.g. presentation of the DEGAM curriculum [13], milestones [27].<br>h) Exams: What must ÄIWs know at the end of their advanced specialty training? Reference to EPA	80	KW

Attachment 1: Content and time schedule of the train the trainer seminar, division of topics between KW and LÄK

Recruiting of ÄiW	Interactive collecting – mutual exchange of experience, presentation of platforms, job exchange of LÄK	15	LÄK
Drafting the contract **– what do I have to consider when hiring ÄiW?**	Small group work on cases, topics: recognition of periods of further training, drafting of contracts, interruption of further training; discussion in plenary, reference to FAQ of KV, IPAM funding, further legal requirements	25	LÄK
Closing Day 1<br>Open questions	Write down the most important findings of the day	10	KW
**Joint dinner**			
**Saturday 9 a.m. – 4 p.m.**			
Arrival, greeting	Collect the most important findings of day 1, clarify open questions, Relate to common thread	10	KW
**Common thread: the ÄiW is in the practice – what tasks do the specialty trainers have during this time?**			
Feedback	Theoretical introduction, interactive collection of feedback rules and completion by lecturers	20	KW
Conversations with ÄiW	Three small groups, each group works on three cases - rotation of the groups, individual break design	180	KW
**Break (60 min)**			
Review morning	Collect new findings	5	KW
**Statement of ÄiW: what do we wish for?**	Lecture and discussion	15	ÄiW/KW
**Tips of other licensed trainers**	Presentation and completion of innovative ideas from other licensed trainers for postgraduate specialty training	10	KW
Difficult cases	Work in small groups on cases, topics: interruption of postgraduate specialty training, deputizing by ÄiW, legal issues, discussion in the plenum	30	LÄK
**Break (15 min)**			
**Common thread: the period of postgraduate specialty training comes to an end**			
**Documentation of postgraduate specialty training**	Creation of certificates, documentation in the logbook, specifications, frequent errors - lecture and work in small groups with examples	30	LÄK
**Open questions, presentation of further material**		15	KW
**Transfer – What exactly do I intend to do**?	Letter to myself, name concrete goals	5	KW
Feedback	Headline for the event or 5-person-method or other activating method, focus: What do I bring to my ÄiW? What do I bring for myself?	15	KW

Attachment 1 to: *Sonntag U, Koch A, Bayer G, Heintze C, Döpfmer S. Train the trainer course for general practice trainers in ambulatory care: the Berlin model. GMS J Med Educ. 2020;37(3):Doc28. DOI: 10.3205/zma001321*
Online available from: https://www.egms.de/en/journals/zma/2020-37/zma001321.shtml

Evaluation, conclusion	Completion of evaluation sheets, oral feedback	10	KW und LÄK
